# Abstract of Professor Heim’s Paper on the Identity of Small-Pox and Cow-Pock

**Published:** 1842-07-23

**Authors:** 


*Abstract of Professor Heim's Paper on the identity of Small-pox and Cow-pock.*—The first notice of Dr. Gassner's experiments is to be found in No. 67 of the "Salzburgher Medic. Zeitg., 1807," in the following terms:—

"*Günzburg.*—Dr. Gassner has inoculated several cows with small-pox lymph, and eleven of the cows have taken the cow-pock. With the matter thus obtained, four children of a curate were vaccinated, and a very fine cow-pock ensued." In the year 1807, an inquiry was held by order of the Minister of the Interior on the announcement just alluded to. Dr. Gassner himself was examined, and declared that in May, 1801, he had made the experiments mentioned. No effect was produced on ten cows; the eleventh he inoculated with the most perfect success. He described the progress of the disease as follows:—On the second, third, and fourth days, nothing was observed in the points where the matter had been introduced; on the fifth day, however, a small red point formed over each; on the sixth, a small transparent vesicle; on the seventh, the vesicle was converted into a bulla *(blase)*, which was surrounded with a reddish circle on the eighth and ninth days; on the tenth day, the whole of the bulla was of a bluish pearl colour, and the lymph was, on this day, taken from it. On the eleventh, the udder was swollen, febrile symptoms set in and continued to the twelfth, when the tumefaction of the udder abated; the pustule was now of a whitish colour. On the thirteenth, the animal began to eat, and there was a depression in the middle of the pustule, which turned into a yellow crust on the fourteenth. From the fifteenth to the sixteenth, the crust became brown, then darker, and remained on to the twenty-eighth day.

Dr. Gassner vaccinated with the lymph several children in the rectory-house at Riedheim, and from them, seventeen others at Schönenberg. The appearances of the pock were perfect.

These circumstances were examined into by the commission of inquiry, who came to the conclusion that the facts averred by Dr. Gassner were a pure fiction.

On the other hand, Professor Heim inquired minutely into all the circumstances of the case, and says he is convinced of the variolation of the cow, and of the transmission from her of a disease similar to vaccinia.

In addition to the evidence collected from the surviving children of the curate, vaccinated by Dr. Gassner, of a contemporary witness, the schoolmaster of Nübling, Professor Heim cites a letter addressed by Dr. Gassner to Dr. V. Ehrhart, and published in his "Sammlung von Beobactungen, 1. B. 1. H. Nürnberg, 1803, s. 94." The following is an extract from this letter:—"I have endeavored to ascertain by experiment the nature of the vaccine lymph. The report that I have inoculated several cows with small-pox is true; the result on the eleventh cow was perfect, and fully bore out the ideas which I had formed from analogy; I vaccinated with the lymph obtained from this cow the four children of a curate at Riedheim, and a beautiful cow-pock was produced. The matter with which I succeeded in producing the disease in the cow was taken from a child labouring under malignant small-pox at Zügen." The letter bears date, Günzberg, April 26, 1802.—***Prov. Med. Jour. May*** 21, 1842.

